# Multimodal explainable AI predicts upcoming speech behavior in adults who stutter

**DOI:** 10.3389/fnins.2022.912798

**Published:** 2022-08-01

**Authors:** Arun Das, Jeffrey Mock, Farzan Irani, Yufei Huang, Peyman Najafirad, Edward Golob

**Affiliations:** ^1^Secure AI and Autonomy Laboratory, University of Texas at San Antonio, San Antonio, TX, United States; ^2^UPMC Hillman Cancer Center, University of Pittsburgh Medical Center, Pittsburgh, PA, United States; ^3^Cognitive Neuroscience Laboratory, University of Texas at San Antonio, San Antonio, TX, United States; ^4^Department of Communication Disorders, Texas State University, San Marcos, TX, United States

**Keywords:** self-supervised, multimodal, deep learning, machine learning, stuttering, EEG, facial expression, disfluency

## Abstract

A key goal of cognitive neuroscience is to better understand how dynamic brain activity relates to behavior. Such dynamics, in terms of spatial and temporal patterns of brain activity, are directly measured with neurophysiological methods such as EEG, but can also be indirectly expressed by the body. Autonomic nervous system activity is the best-known example, but, muscles in the eyes and face can also index brain activity. Mostly parallel lines of artificial intelligence research show that EEG and facial muscles both encode information about emotion, pain, attention, and social interactions, among other topics. In this study, we examined adults who stutter (AWS) to understand the relations between dynamic brain and facial muscle activity and predictions about future behavior (fluent or stuttered speech). AWS can provide insight into brain-behavior dynamics because they naturally fluctuate between episodes of fluent and stuttered speech behavior. We focused on the period when speech preparation occurs, and used EEG and facial muscle activity measured from video to predict whether the upcoming speech would be fluent or stuttered. An explainable self-supervised multimodal architecture learned the temporal dynamics of both EEG and facial muscle movements during speech preparation in AWS, and predicted fluent or stuttered speech at 80.8% accuracy (chance=50%). Specific EEG and facial muscle signals distinguished fluent and stuttered trials, and systematically varied from early to late speech preparation time periods. The self-supervised architecture successfully identified multimodal activity that predicted upcoming behavior on a trial-by-trial basis. This approach could be applied to understanding the neural mechanisms driving variable behavior and symptoms in a wide range of neurological and psychiatric disorders. The combination of direct measures of neural activity and simple video data may be applied to developing technologies that estimate brain state from subtle bodily signals.

## 1. Introduction

For the brain, as in life, change is a constant. The main goal of cognitive neuroscience is to reveal the mechanisms of how brain dynamics, in terms of spatial and temporal activity patterns, relate to behavior. The problem of understanding brain dynamics has been approached from multiple directions, including neural recordings at the levels of single units and local field potentials (Buzsáki et al., 2012), and macro-level far field recordings using EEG and MEG (Makeig et al., 2004; Stam, 2005). Epilepsy patients with intracranial electrodes have provided detailed information about human neural dynamics that is unavailable from non-invasive methods (Mukamel and Fried, 2012). Neuroimaging studies have revealed the slow resting dynamics of large neural networks at rest that are recapitulated, to some degree, during behavioral tasks (Smith et al., 2009; Laird et al., 2011). Lastly, computational modeling draws connections between brain activity at different spatial and temporal scales and tests general principles that may account for patterns of activity and their relations to behavior (Sejnowski et al., 1988; Kelso, 1995).

With the exception of epilepsy, studies of patients with neurological disorders have not played a major part in understanding brain dynamics. In many other areas, neuroscientists have learned much from neurological patients, particularly when the disorder causes exaggerated instances of typical behavior. Patient HM is a classic example. Following neurosurgery to treat epilepsy, HM lost the ability to remember his personal experiences—a tragedy that helped researchers understand the crucial role of the hippocampus in episodic memory (Scoville and Milner, 1957). In the realm of language, the aphasic patient “Tan” provided the first convincing evidence that an intact left inferior prefrontal cortex is vital for normal speech production (Broca, 1861). Such patients cast normal behavior into sharper relief. However, in most neurological patients, the extreme impairment is consistent—HM's amnesia and Tan's aphasia were unrelenting. This poses a challenge for using neurological disorders to understand brain-behavior dynamics because the behavioral impairment is generally static (i.e., exists within a small range of variability).

One common neurological problem with a behavioral impairment that is both extreme and dynamic is persistent developmental stuttering (Bloodstein et al., 2021). People diagnosed with persistent developmental stuttering are adults who began to stutter as children with no known neurological or emotional trauma preceding stuttering onset (Bloodstein et al., 2021). There are several reasons why adults who stutter (AWS) present a good opportunity to study relations between brain dynamics and behavior. First, unlike most brain disorders, AWS is within the normal range in terms of cognition, emotion, socialization, education, professional and socioeconomic achievement, and quality of life (Craig et al., 2009; McAllister et al., 2013; Guitar, 2014). Stuttering is also a highly selective behavioral impairment, which avoids confounds that would need to be controlled in other brain disorders such as general impairments in attention, memory, lucidity, and intellect. Although at the group level, there are subtle anatomical differences in AWS relative to matched controls (Cai et al., 2014; Chow and Chang, 2017; Chang et al., 2019), the brains of individual AWS are clinically in the normal range. Against a backdrop of near-normalcy, AWS sometimes has obvious behavioral abnormalities when speaking. The stark contrast between fluent and stuttered speech is useful for defining neural mechanisms of dynamic behavior if one assumes proportionality between the magnitude of behavioral differences and the magnitude of differences in the neural signals driving the behaviors. Lastly, speech outcomes in AWS vary on a time scale of seconds—fluent speech is occasionally punctuated by stuttering, which then reverts back to fluent speech. The relatively brief episodes of stuttered speech act as an internal control for general states that change over longer time periods (minutes to hours), such as fatigue or anxiety. That is, continuous speech output in AWS can alternate between fluent and stuttered on the order of seconds, while states such as fatigue and anxiety last much longer, on the order of minutes to hours.

In prior studies, EEG that was recorded when AWS and fluent controls prepared to speak had group differences in specific evoked responses to stimuli and ongoing oscillations differed among groups (Maxfield et al., 2012; Mock et al., 2015, 2016). EEG activity during speech preparation also strongly predicted individual differences in stuttering severity among AWS (Daliri and Max, 2015; Mock et al., 2015, 2016). Identifying neural correlates of individual differences at the levels of group (AWS vs. fluent controls) and individuals (within AWS) are the first steps to understanding brain-behavior dynamics in AWS. This project takes the next step by examining how trial-by-trial brain dynamics within the same person relate to upcoming speech that is either fluent or stuttered. To achieve this goal, we will apply multimodal AI methods that use EEG and facial video data.

A deep learning model named AlexNet (Krizhevsky et al., 2012), introduced in 2012, achieved state-of-the-art performance in the 1,000-class ImageNet image classification problem. This led to a paradigm shift in deep learning parallel computation relative to the “AI winter” of 1990s and early 2000s. Ever since, the phrase “ImageNet moment” is used to describe the success of deep learning algorithms and the rapid adoption of deep learning algorithms in a variety of applications in the sciences, engineering, and healthcare. Jirayucharoensak et al. (2014) introduced fully connected neural network architectures to EEG analysis, and they discovered feature correlations to emotions experienced by participants watching music videos. By classifying non-stationary EEG states in terms of valence (negative, neutral, positive) and arousal metrics, Jirayucharoensak et al. explored how EEG-based deep-learning algorithms can improve automatic emotion recognition. Recent deep learning algorithms improve upon previous blind classifiers, which are designed to be robust and informative, and often focus on patient specific information (Dissanayake et al., 2021) and online decision-making (Cho et al., 2021) to improve real-world implementations.

Binary classification of brain states using deep learning algorithms has been used to learn the small nuances in EEG data to predict the sex of subjects (Van Putten et al., 2018), which is trivial for humans with visual or auditory information but extremely hard without them. With the proper cognitive experimental design, researchers have introduced deep learning algorithms that can predict future brain states from pre-event sensory data. In Idowu et al. (2021), the authors introduced an LSTM-SAE model to predict the future motor intention of users undergoing visual stimuli. However, neuroscience problems are inherently multimodal, relying on unimodal information could skew results (Abrol et al., 2019).

The face is awash in useful information about cognitive and emotional states. This includes various voluntary and involuntary muscle movements due to facial expressions (Barrett et al., 2019), eye movements and direction of gaze (Grossmann, 2017), blinking (Stern et al., 1984; Jongkees and Colzato, 2016), and pupil dilation controlled by muscles within the eye (Bradley et al., 2008). Emerging work suggests that voluntary behaviors, such as speaking, are accompanied by subtle micro movements in the face (Aouayeb et al., 2019; Rundo et al., 2019; Yang et al., 2021) and body (Cho et al., 2021). Micromovements in the body are subtle changes in individual muscle groups and have been used to study speech and behavior since the 1970s (Condon and Sander, 1974). Micromovement data from external body sensors enables fined-grained decision-making and superior understanding of disorders by using external body sensors (Bifulco et al., 2011), RFID tags (Colella et al., 2021), etc. However, micromovements are often overlooked in clinical research because they are difficult to identify and measure and are challenging to interpret because the neural mechanisms and accompanying information processing are unclear.

Relatedly, facial microexpressions are brief, subtle involuntary muscle movements from the upper and lower facial regions (Ekman, 2009; Verma et al., 2019). Microexpressions convey descriptive information that may vary among different emotional states (Ekman et al., 1983) and cultural differences (Ekman, 1992). Note, however, that the facial expressions have a rich and controversial history of indexing emotions (Darwin, 1872; Barrett et al., 2019). The successful encoding of facial muscle movement patterns as facial Action Units (AU) is based on the Facial Action Unit Coding System (FACS) (Ekman et al., 1983; Ekman and Rosenberg, 2005). Recent research have shown the ability to use FACS as a way to quantify human attention and affect (Lints-Martindale et al., 2007; Hamm et al., 2011), and pain (Kunz et al., 2019). Relatedly, AI algorithms using EEG signals as inputs can distinguish among cognitive states and are relevant to understanding neurological disorders such as Alzheimer's disease (Zhao et al., 2019) and Parkinson's disease (Oh et al., 2018).

In this work, we present a multimodal deep learning algorithm capable of relating brain activity (EEG) and facial microexpressions to predict future stuttering events from pre-speech multimodal data. Earlier studies explored either facial expression data (Das et al., 2021) or EEG signal information (Myers et al., 2019) to predict future stuttering events. However, no studies have examined EEG and video (facial movement) modalities together. We believe that using facial muscle activity and EEG data for interpretable machine learning models will provide insight into how peripheral measures of microexpressions relate to internal neurocognitive states. Here, we trained the deep model on a controlled experimental study dataset. The goal of this study was to detect EEG and facial muscle activity signals that precede vocalizations and can jointly predict fluent vs. stuttered speech outcomes.

To achieve this goal, we built a novel neural network capable of faithfully classifying trial-by-trial dynamics of EEG and facial activity that can predict upcoming speech behavior. To evaluate the method, we explore the dynamics of differing EEG states during speech preparation and facial muscle movements of AWS. We propose to use self-supervised multimodal architectures that can learn the temporal dynamics of both EEG and facial muscle movements during speech preparation. We show that our proposed algorithm can learn to predict upcoming fluent vs. stuttered speech from a small amount of labeled data from AWS who perform several variations of a speech preparation task. By using a multimodal Shapley deep learning model explainer, we found that left temporal and right frontal brain activity, as well as upper and lower facial microexpression at both early and late stages of speech preparation anticipate stuttering.

## 2. Materials and methods

### 2.1. Related work

#### 2.1.1. AWS studies using EEG and facial activity data

Prior EEG work found a host of differences in EEG signals during speech preparation in groups of AWS vs. fluent controls (Maxfield et al., 2012; Mock et al., 2015, 2016). Moreover, EEG activity during speech preparation covaries with individual differences in stuttering severity (Daliri and Max, 2015; Mock et al., 2015, 2016). In a pioneering work on stuttering, Kelly et al. (1995) used electromyography to describe atypical tremor-like facial activity patterns in children who stutter. Multimodal facial and EEG data have been later used to summarize the vigilance and empathetic nature of infants (Field et al., 1998). More recently, machine learning based algorithms have started to fuse multimodal data including EEG and spontaneous facial expressions to predict affective states (Sun et al., 2020).

#### 2.1.2. Multimodal deep learning

In multimodal deep learning, neural networks are used to integrate, fuse, and learn complementary representations from multiple input domains (Ngiam et al., 2011). Recent work has successfully fused images and text (Abavisani et al., 2020), detected adverse weather by combining different types of sensor information (Bijelic et al., 2020), estimated the 3-D surface of faces (Abrevaya et al., 2020), and combined information from multiple drug and diagnosis domains (Linden et al., 2021).

#### 2.1.3. Self-supervised learning

Self-supervised learning algorithms learn meaningful representations from unlabeled data by creating pretext tasks based on pseudo-labels (Sheng et al., 2020). After defining a custom pretext task, a deep learning model is then optimized to predict the pseudo-labels. After successful pretext task training, the final downstream task is carried out on the available labeled data. Currently, self-supervised learning is used to learn temporal correspondences in videos (Tschannen et al., 2020), disfluency detection from text to improve annotation (Wang et al., 2019), and has many applications to reduce the time needed to label data.

#### 2.1.4. Estimating brain and facial muscle activity using deep learning

Human brain activity can be monitored in real time with EEG, which allows researchers to better understand how humans see (Spampinato et al., 2017) and think from facial behavior (Pilz et al., 2020), emotions, and whether what we are thinking is deceptive or not (Gupta et al., 2019). The human face exhibits both voluntary and involuntary muscle activity, and analysis of facial movements can be used to assess and diagnose various diseases (Jiang et al., 2020). A common way to define facial muscle movements is by encoding their activity as facial action unit (AU) patterns (Friesen and Ekman, 1978).

Research shows that people who stutter often have “secondary behaviors” such as eye-blinking, sudden jaw movements, or other involuntary movements of the head or limbs while speaking (Prasse and Kikano, 2008). We hypothesized that AWS may demonstrate more subtle microexpressions before speech onset. Numerous studies describe relationships between upper and lower facial muscle movements and affect (Ross et al., 2007; Wang et al., 2013), as well as relations to concurrent audio recordings of speech (Meng et al., 2017).

#### 2.1.5. Automated estimation of stuttering disfluency

Stuttering is a neurological speech disorder which affects the normal flow of speech with word or syllable prolongations, silent blocks, and/or part-word repetitions. The neuronal speech networks of AWS fluctuate between brain states that are conducive to either fluent or stuttered speech output. When stuttered speech occurs, it appears on the initial sound/syllable 90% of the time (Sheehan, 1974). Hence, speech preparation is an important time period that is both free of speech artifacts and should contain signals that can distinguish upcoming speech as either fluent or stuttered.

Recent work found that pre-speech EEG data can be used to classify stuttering events (Myers et al., 2019). Prediction of stuttering using pre-speech facial muscle movements has been explored by Das et al. (2021). In Das et al. (2020)'s study, the authors did not address the interplay between facial expressions and cognitive states and ignored the multimodal nature of neuroscience research. To the best of our knowledge, there are no published studies that used both facial muscle movements and EEG activity as inputs for predicting stuttering.

### 2.2. Problem formulation

One of the main goals of deep learning optimizations is to learn a function $f:X\to {\mathbb{Z}}^{D}$ to represent inputs *x* as feature representations *z* = *f*(*x*). Previous research studies have shown the successful use of deep learning algorithm to predict the cognitive states of AWS by inputting facial action units (Das et al., 2021) or EEG signals (Myers et al., 2019). However, in domains such as neuroscience, studies involving behavior often require a temporal understanding of multiple modalities. Hence, feature representations of individual modalities might not be beneficial to understand certain neurological disorders. There is a need to expand the input space X such that representations are now f(X) where $X=x_{1},x_{2},\cdots \;,x_{m}$ for *m* modalities. Here, independent features from each modality could benefit from each other during optimization.

**Figure 1:**
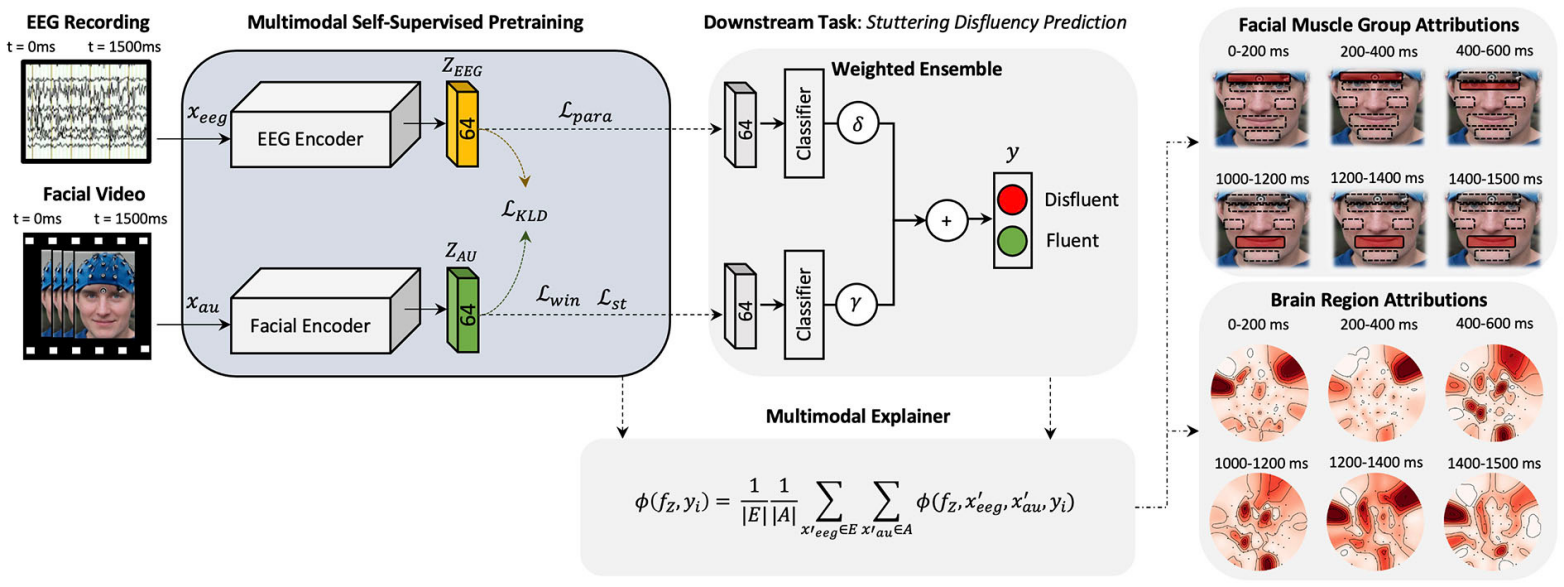
A high-level architecture of the proposed explainable multimodal self-supervised approach is presented. Two CNN models are pre-trained with self-supervision and latent space combined to form a multimodal architecture for feature-wise ensemble fusion. Multimodal explanations are generated (one explanation map for each input) using the proposed multimodal shapley explanation method and the highest attributing features are presented.

To improve the decision-making accuracy and robustness of existing stuttering classifiers and to demonstrate the importance of multimodal deep neural networks in neuroscience problems, we plan to combine facial AU data representations presented in Das et al. (2021) and EEG data representations presented in Myers et al. (2019) under a common multimodal deep learning framework. Here, we learn a multimodal function $f:X\to {\mathbb{Z}}^{D}$, where $X=\left\{x_{au},x_{eeg}\right\}$ and ℤ = {*Z*_*AU*_, *Z*_*EEG*_}. Deep learning algorithms require large number of labeled data to optimize the model parameters. Recently, self-supervised deep learning algorithms are being used to mitigate the big-data requirements of deep learning algorithms by pre-training these large networks by defining pretext tasks. Hence, to learn the multimodal representations from limited labeled data available, we explore a self-supervision method to learn facial muscle dynamics and internal cognitive states from unlabeled data of AWS (Figure 1) such that embeddings *Z*_*AU*_ and *Z*_*EEG*_ learns dense feature representations of the corresponding inputs. These pre-trained networks can then be combined to learn ℤ = {*Z*_*AU*_, *Z*_*EEG*_} under a multimodal framework to detect neurological diseases by sharing features from both modalities, ultimately improving the robustness and accuracy of model predictions.

### 2.3. Self-supervised multimodal explanations

Inputs to multimodal networks often have correlations which also helps the network to learn features between the inputs, for example, an image and a related caption (Park et al., 2018). As stated above, to learn the dynamics of the internal cognitive states and external muscle movements, we extend the work done in Myers et al. (2019) and Das et al. (2021) and plan to learn meaningful representations from both modalities in a combined multimodal training paradigm. Figure 1 illustrates a high-level diagram of the multimodal architecture. Here, we develop two Convolutional Neural Networks (CNNs) *H*_*EEG*_ and *H*_*AU*_ parallel to each other. This creates dense representations of corresponding EEG and AU trial inputs which are influenced by each other. We then pre-train this multimodal network using self-supervised training methods. Before proceeding further, we will introduce the cognitive experimental study as the basis of future explanations.

#### 2.3.1. AWS speech study dataset

A multimodal behavioral dataset from a group of AWS subjects was introduced in Das et al. (2020) based on an ongoing study with a unique, longitudinal data set of speech, EEG, and video data in adults who have stuttered since childhood. We requested the authors and gained access to the dataset which was being collected during a 3-year NIH grant study. Every subject (n=7) came to the laboratory over the course of 2 months for 3–5 sessions (400 trials/session) of data collection. Both facial videos and EEG were collected from individual subjects, while they read the experimental stimuli, for each study day. Facial muscle movement information was quantified by extracting facial AU vectors from the collected facial videos. Hence, facial EMG sensors were not required to study facial muscle movements and EMG signals are not used in this study. Pre-processing of collected facial videos and EEG signals are detailed in Section 3. All studies were done under strict protocols of the Institutional Review Board and the Declaration of Helsinki. All subjects were self-reported to have developmental stuttering prior to the study and were diagnosed by a Speech-Language Pathologist (SLP) for verification.

##### 2.3.1.1. Speech-motor experiment paradigms

**Figure 2:**
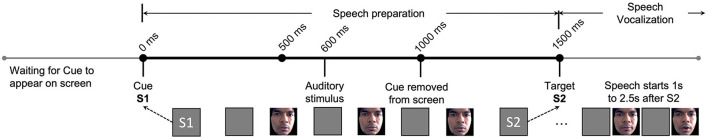
Experimental paradigms and their timing diagram is illustrated. Here, S1 represents the Cue where the trial begins. S2 represents the Target where a vocal response from subject is requested. The EEG and facial activity inputs to our proposed machine learning model are strictly limited to between S1 and S2 during the speech preparation phase. The speech vocalization phase is immediately after S2, and speech usually happens 1–2.5 s after S2 based on the individuals stuttering severity. Changes in the facial muscles of the first author are illustrated as an example, shared after approval and consent.

The subjects were seated in a sound isolating booth 50 cm away from a monitor in front of them. A small delay (“S1-S2” paradigm) between speech preparation and speech execution allowed researchers to study the brain activity that preceded a stuttered or fluent speech vocalization and correlate it with corresponding facial movements, as illustrated in Figure 2. S2 onset always occurs 1,500 ms after S1 onset. Both facial video and EEG data were simultaneously collected. Facial videos are converted to facial muscle movements by quantifying the small changes in facial muscles as facial AU vectors. The machine learning model utilizes this synchronized AU and EEG pair during the speech preparation phase, clipped between S1 and S2 (1,500 ms in total), to learn multimodal pre-speech representations that could predict future stuttering behavior. As seen in Figure 2, speech vocalization phase beings only after S2, and the speech output of AWS typically start between 1 to 2.5 s from the onset of S2. This ensures that the facial activity does not overlap with stuttering or speech events.

Four different variations of the S1-S2 paradigm were used to study the impact on predictability when the subject either has a specific speech plan in memory or does not. In the “Word-Go” (WG) paradigm, the subject is shown a “pseudoword pair” on the monitor at S1 and a “!!!” symbol at S2. S1 tells the subject exactly what needs to be spoken after S2 (“!!!”) onset. This paradigm adds extra phonological information between S1-S2 and is called the “memory task.”

In the “Cue-Word” (CW) paradigm, the subject is shown the “+” symbol at S1 and the pseudoword pair at S2. Here, the subject does not know the word to speak until S2 onset. Hence, we call this variation the “non-memory task.” To study the impact of auditory cues in the speech-motor plan, we also presented a 1,000 Hz pure tone beep at 600 ms after S1 for both the WG and CW paradigms. We called these paradigms ‘Word-Auditory-Go' (WAG) and ‘Cue-Auditory-Word' (CAW). The four paradigms are further summarized in Table 1.

**Table 1:** Design considerations for the self-supervised pretext tasks for AU transformations and EEG cognitive tasks.

**Cognitive experiment pretext task**		
**Cognitive experiment pretext task**	**Label**	**Description**
Word in memory (WG)	0	Word at S1 (0 ms), “!!!” at S2 (1,500 ms).
Word not in memory (WAG)	1	Word at S1 (0 ms), Tone at 600 ms, “!!!” at S2 (1,500 ms).
Word not in memory (CW)	2	“+” at S1 (0 ms), Word at S2 (1,500 ms).
Word not in memory + audio cue (CAW)	3	“+” at S1 (0 ms), Tone at 600 ms, Word at S2 (1,500 ms).
**Signal transformation pretext task**		
Signal transformation	Parameters θ_*st*_	
Scaling	{0.25, 0.5, 1.25, 1.75}	
Gaussian noise	{0.1, 0.25, 0.5, 0.75, 0.9}	
Zero fill	-	
None	-	

##### 2.3.1.2. Stimuli for speech trials

Participants were presented with a random combination of two non-words as stimuli to be read aloud. All non-words phonetically mimicked English words did not have any meaning and started with a consonant-vowel. Non-word pairs were used because preliminary work found them useful for attaining an equal ratio of fluent and stuttered trials (Myers et al., 2019). For each session a custom MATLAB script randomly selected 400 non-word pairs for presentation, ensuring that the first and second non-words in the pair did not begin with the same consonant.

##### 2.3.1.3. Coding of speech trials

Video recordings of each trial were coded as either fluent, stuttered, normal disfluency, or missed. A trial was labeled as stuttered if the participant produced a stuttering-like disfluency (Bloodstein et al., 2021) on either (or both) non-words. Stuttering-like disfluencies include repetition of a sound, syllable, or part of the word (e.g., c-c-c-cookie); prolongation of a sound (e.g., lllllllllight); or a silent block. (e.g., T- - - - - -all). Trials with any other type of disfluency (i.e., hesitation or interjection) or trials where the participant did not attempt were excluded from the analysis.

#### 2.3.2. Self-supervised pre-training of *H*_*AU*_ and *H*_*EEG*_

Self-supervised learning algorithms have grown in interest as a way to learn meaningful representations from unlabeled data by creating pretext tasks based on pseudo-labels. Once an auxiliary learning task is defined, called a pretext task, self-supervised training can force the network to learn data features without human-annotated labels. These learned features could be useful for further downstream optimization on a small number of human-labeled data. In our case, self-supervision could dramatically reduce the need to label individual facial AU and EEG trials as fluent or stuttered while also learning meaningful representations related to stuttering disfluency.

Self-supervision of facial AU data carried out by Das et al. (2020) focused solely on the facial microexpressions. However, the cognitive states leading to the facial microexpressions have equal, if not more, impact on learning rich representations. Hence, we propose to force the latent representation *Z*_*AU*_ of the facial encoder to be closer to latent representations of EEG encoder *Z*_*EEG*_ using Kullback Leibler (KL) Divergence loss LKLD during optimization. Now, to learn meaningful representations, we design the EEG pretext task based on existing experimental metadata such that the *Z*_*AU*_ learn not only about the facial microexpressions but also about the different cognitive contexts based on whether a word is in memory or not and the impact of the auditory stimulus.

##### 2.3.2.1. Pre-training of *H*_*EEG*_

Our cognitive experimental design generates experimental metadata which holds information about the different cognitive experiments conducted as summarized in Table 1. We use this metadata information as pseudo-labels for pre-training *H*_*EEG*_ to distinguish between the different cognitive experiments. This reinforces the latent space with cognitive states associated with having a word in memory and the impact of hearing an auditory stimulus during the phonological phase and attention. Now the goal of *H*_*EEG*_ during pre-training is to predict the paradigm information *Y*_*para*_ successfully. Loss function for the training, using cross entropy, now becomes


(1) $$\begin{array}{l} L_{para}=-\overset{4}{\underset{para=1}{\sum }}y_{para}\log\left(p_{para}\right) \end{array}$$

where *y*_*para*_ is the true paradigm label and *p*_*para*_ is the predicted output by *H*_*EEG*_.

##### 2.3.2.2. Pre-training of *H*_*AU*_

Building upon the work done by Das et al. (2021), to improve the learning performance by including representations for sudden jerks, partial or full occlusions, we apply signal transformations on a small 100 ms time-window *w*. Gaussian noise, scaling, and zero-filling with predefined parameters of augmentation are explored. Here, each transformation, as in Table 1, has a neurological relation. For example, Gaussian noise can model partial occlusions and scaling can model sudden jerks. Now, we define two loss functions, first $L\text{st}\left(w,\theta_{st}\right)$ to find the correct signal transformation *st* applied with a parameter θ_*st*_ to window *w*, and second Lwin(w) to find the window *w* in which the transformation was applied. To learn disentangled features from the upper and lower face, we always chose either the upper or lower facial regions for *w* and never both together.


(2) $$\begin{array}{l} L\text{st}\left(w,\theta_{st}\right)=-\log P\left(\tilde{st}=st|m\left(w,\theta_{st}\right)\right) \end{array}$$

(3) $$\begin{array}{l} L\text{win}\left(w\right)=-\log P\left({ỹ}_{win}=y_{win}|w\right) \end{array}$$

where *y*_*win*_ is the label for window *w*, ỹ_*win*_ is the prediction, and *P*_*win*_ is the probability of picking the correct window.

##### 2.3.2.3. Total loss function

Total loss for the self-supervised pre-training of multimodal representations now becomes


(4) $$\begin{array}{l} \begin{array}{c} L=L\text{para}+\alpha \cdot L\text{win}\left(w\right)+\beta \cdot L\text{st}\left(w,\theta_{st}\right)+\\ L\text{KLD}\left(Z_{AU},Z_{EEG}\right) \end{array} \end{array}$$

where α and β are used to balance the loss term. Together, the optimization helps to learn spatio-temporal relationships in time-series AU and EEG features, which is fundamentally different from other image-based self-supervision algorithms.

#### 2.3.3. Multimodal downstream task optimization

For the final task of predicting near future stuttering, embeddings *Z*_*EEG*_ and *Z*_*AU*_ can be combined using a weighted ensemble method using non-parametric multipliers δ and γ. Here, the final result will be a Sigmoid threshold on the weighted classifier results of both *H*_*EEG*_ and *H*_*AU*_.


(5) $$\begin{array}{l} y=Sigmoid\left(\delta *{ỹ}_{eeg}+\gamma *{ỹ}_{au}\right) \end{array}$$

where ỹ_*eeg*_ and ỹ_*au*_ are predicted stuttered vs. fluent labels of *x*_*eeg*_ and *x*_*au*_ inputs.

##### 2.3.3.1. Multimodal shapley explanations

**Figure 3:**
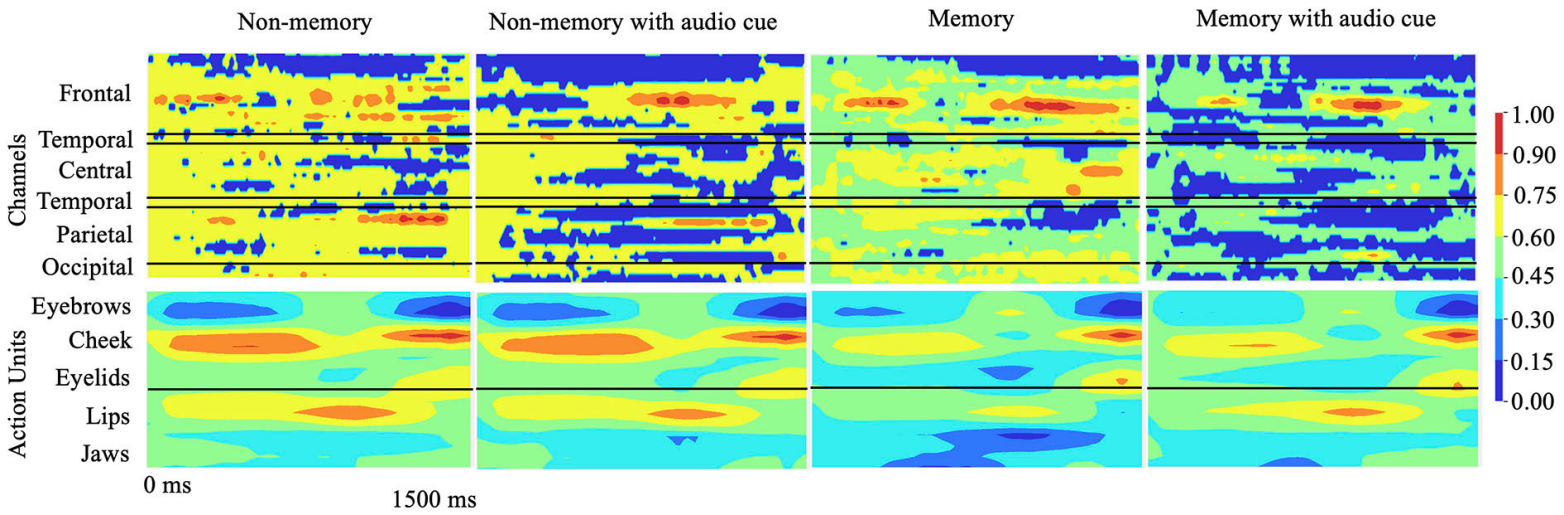
Qualitative results: 2D explanation-map-pairs generated for different cognitive experiments for multimodal inputs (AU and EEG) using the proposed multimodal DeepSHAP algorithm (normalized) are illustrated. Spatio-temporal impact of EEG or face AU toward disfluency can be understood using this temporal map-pair. The red region shows the highest impact toward stuttering prediction while the blue region shows the least impact.

To explain the cognitive states and facial muscle movements with the highest correlations to stuttering events, we formulate the explainability analysis to generate one explanation map each for individual modality (EEG and AU) and find dependencies between the highest attributing features from both modalities as illustrated in Figure 3.

Consider the original multimodal prediction model as *f*. The task to explain is *f*(*x*_*eeg*_, *x*_*au*_) based on the two inputs. Considering *f*_*Z*_ as the embedding layer of *f*, a linear approximation of the combination Shapley values for each modality can be calculated based on DeepLIFT multipliers (Lundberg and Lee, 2017; Shrikumar et al., 2017), *m*, such that


(6) $$\begin{array}{l} \phi_{i}\left(f_{Z},y\right)\approx m_{y_{i}f_{Z}}\left(y_{i}-E\left[y_{i}\right]\right) \end{array}$$

where ϕ_*i*_ corresponds to the attribution of individual features in each modality. An average marginal contribution of features based on local feature attributions of both EEG and AU can be calculated citing the additive nature of Shapley explanations, with feature removals of corresponding inputs $x_{eeg}^{\prime }$ and $x_{au}^{\prime }$ influence ϕ_*i*_(*f*_*Z*_, *y*) such that


(7) $$\begin{array}{l} \phi \left(f_{Z},y_{i}\right)=\frac{1}{\left|E\right|}\frac{1}{\left|A\right|}\underset{x_{eeg}^{\prime }\in E}{\sum }\underset{x_{au}^{\prime }\in A}{\sum }\phi \left(f_{Z},x_{eeg}^{\prime },x_{au}^{\prime },y_{i}\right) \end{array}$$

Thus, the output of DeepSHAP are two explanation maps $E\left(x_{au}\right)\in {\text{R}}^{17\times 87}$ and $E\left(x_{eeg}\right)\in {\text{R}}^{62\times 192}$ with positive and negative correlations of each AU and EEG toward the final classifier decisions. Since explanations are generated based on a reference, we fix the reference to be sampled from the same population based on the subject, session, and paradigm under study.

## 3. Experiments

In this section, we detail our data processing pipeline, experimental training setup, hyper parameters, and evaluation metrics.

### 3.1. Data processing and statistics

From the cohort of AWS subjects, a Speech Language Pathologist (SLP) selected 7 AWS subjects and labeled individual trials as either stuttered or fluent. This generated 3,515 labeled trials for the final downstream training. To pretrain our self-supervised model, we generated a pretraining dataset of 69,000 trials by applying the pretext transformations presented in Table 1 to the available unlabeled face AU and EEG data.

EEG data were recorded using a standard 10-20 montage with 64 channel electrode cap (60 scalp Ag/AgCl electrodes impedances ≤ 10 kΩ). Eye movements were monitored using four electrodes, two lateral to each eye and one each above and below the left eye. The data collected was digitized at 1,000 Hz with a DC-100 Hz bandpass filter using Curry 7 Neuroimaging Suite (Compumedics Neuroscan, Charlotte, NC). Independent Component Analysis (ICA) was carried out to remove movement artifacts from the EEG signals. Note that facial expressions using video information already capture muscle movements. The result of ICA was normalized by recording specific constant (per session). The resulting EEG data were resampled to 128 Hz. Now, for each EEG trial *x*_*eeg*_, we have temporal steps *N*_*e*_=192 for 1.5 s of information. We focus on 62 channels of information such that *I*_*e*_=62.

The video data were split into fifty 1,500 ms long video trials per paradigm per study. Video data were sampled at 58 frames/sec (17 ms temporal resolution). The number of frames for each trial *v* is *N*_*v*_=87. Noisy transient AUs associated with head movements (AUs 51-64), some AUs in the upper (nostril dilator AU 38) and lower (lip pucker AU18, bite AU32, suck AU35) facial regions, and some associated with jaws (AU27, 29, 30, 31) were removed to recreate the dataset in Das et al. (2021). Hence, for each trial *v*, we generated an input AU map $x_{au}\in {\text{R}}^{I_{v}\times N_{v}}$, where *I*_*v*_=17.

### 3.2. Network architecture details

We present three variants of SSML architecture. In SSML-A, facial encoder *H*_*AU*_ consists of 4 Convolutional (Conv) layers with {16, 32, 64, 64} kernels, respectively, all shaped 1 × 17 (*I*_*v*_=17 AUs). This is followed by depth-wise (DepthConv) and separable (SepConv) convolutions. Final embedding *Z*_*AU*_ is of 1 × 64 dimensions. In the EEG encoder, *H*_*EEG*_, data extracted from one Conv layer with 16 kernels, all shaped 1 × 62 (*I*_*e*_=62 channels), is passed to a DepthConv layer with 16 kernels and a depth factor 2 to compress the data along the channels. A SepConv layer with 16 kernels is then used to summarize individual feature maps and later flattened to an embedding *Z*_*EEG*_ of 1 × 64 dimensions.

In SSML-B architecture (280k parameters) both *H*_*AU*_ and *H*_*EEG*_ consist of 3 Conv layers with {16, 32, 64} kernels, respectively, all shaped 3 × 3 with max-pooling layers to create embeddings of 1 × 64 in each path. To study the impact of additional layers, SSML-C (317k parameters) has an additional Conv layer with 64 kernels of size 3 × 3 in both branches.

### 3.3. Hyper parameters and training details

The labeled dataset for the downstream task was split equally between stuttered and fluent trials. From this balanced dataset, 10% of the data was separated as a hold-out test dataset including all subjects. From the rest of the available data, we selected 10-folds of training (90%) and validation (10%) data for a 10-fold cross-validation study. We trained all CNN classifiers on an NVIDIA DGX A100 server, using the Tensorflow framework (Abadi et al., 2016), with Stochastic Gradient Descent (SGD) optimizer at an initial learning rate (LR) of 0.01. LR rate halves with every 25 epochs of validation loss plateaus. Batch normalization, a 50% dropout of nodes, and early stopping were applied to curb overfitting.

### 3.4. Evaluation metrics

We evaluate the proposed models using the Area-Under-Curve (AUC), F1 score, and accuracy (same number of stuttered and fluent trials). Additionally, we compare the same performance with Support Vector Machine (SVM) as the downstream classifier.

### 3.5. Statistical significance of explanations

The statistical significance of the multimodal explanations is calculated by carrying out Analysis of Variance (ANOVA) tests. ANOVA is carried out on both *E*(*x*_*au*_) and *E*(*x*_*eeg*_) to study the impact of stutter rate and specific time zones (0–500, 500–1,000, 1,000–1,500 ms) to understand the dynamics of muscle movements of AWS a lot and their internal brain states across time compared with fluent trials.

## 4. Experimental results and discussions

As illustrated in Figure 1, the multimodal downstream task was built using a weighted ensemble of EEG and AU classifiers with non-parametric multipliers δ and γ. With proper tuning of δ and γ, the network learns to not only associate common features but also penalize the under-performing branch. Results tabulated in Table 2 show the consistent performance improvements of self-supervised multimodal learning (SSML) over multimodal fully supervised learning (denoted as FSML). Table 2 also summarizes the usage of non-parametric models such as SVM denoted as SSML-(A,B,C) SVM. The SVM model overfits, resulting in poor performance, due to the highly non-linear behavior of the spatio-temporal embeddings and less amount of labeled data for the downstream task. Tree-based models such as decision trees and random forests also resulted in poor performance. Due to space constraints, we are keeping the results of only the SSML-(A,B,C) SVM models. A comparison with results published in Das et al. (2021) is also provided. We can see a considerable improvement in the accuracy of predicting future stuttering events based on pre-speech data when we use both EEG and facial muscle movement modalities for prediction.

**Table 2:** Multimodal downstream task performance statistics. Here, SSML-* describes the results with pre-trained *H*_*AU*_ and *H*_*EEG*_ following SSML-* architecture with 3 additional dense layers for the downstream task.

**Models**	**AUC**	**F1**	**Acc (%)**
**Our Multimodal (AU and EEG) Methods**			
SSML-A	0.81 ± 0.01	**0.79** **±0.02**	**80.80** **±2.36**
SSML-B	0.80 ± 0.01	0.77 ± 0.02	80.07 ± 2.09
SSML-C	0.77 ± 0.02	0.75 ± 0.03	78.07 ± 2.64
FSML-A	0.79 ± 0.02	0.78 ± 0.02	79.08 ± 2.43
FSML-B	0.78 ± 0.01	0.77 ± 0.02	77.79 ± 1.89
FSML-C	0.75 ± 0.01	0.73 ± 0.01	75.21 ± 2.24
SSML-A SVM	0.51 ± 0.00	0.69 ± 0.03	52.91 ± 3.54
SSML-B SVM	0.52 ± 0.07	0.51 ± 0.02	51.79 ± 7.41
SSML-C SVM	0.50 ± 0.00	0.67 ± 0.04	52.93 ± 4.72
**State-of-the-Art AU Unimodal Method (Das et al.**, **2021****)**			
SS-CNN-A	**0.82** **±0.01**	0.73 ± 0.01	75.27 ± 1.11
SS-CNN-B	0.82 ± 0.01	0.74 ± 0.02	74.83 ± 1.15
SS-CNN-C	0.82 ± 0.01	0.73 ± 0.03	74.53 ± 1.61
FS-CNN-A	0.74 ± 0.05	0.66 ± 0.04	72.76 ± 1.38
FS-CNN-B	0.81 ± 0.02	0.72 ± 0.03	74.53 ± 1.11
FS-CNN-C	0.81 ± 0.04	0.70 ± 0.04	74.63 ± 1.70

FSML-* is fully supervised from scratch. SS-CNN-* and FS-CNN-* represent self-supervised and fully supervised models from Das et al. (2021). Bold values represents the best performing method out of all experiments carried out.

Table 3 summarizes the performance statistics for training *H*_*EEG*_ independently as a unimodal optimizer on the EEG data. Here, SSUL-(A,B,C) denotes pre-training using pretext tasks defined in Table 1 and Unimodal Learning FSUL-(A,B,C) denotes training from scratch. SSUL-A for EEG data performed better than Das et al. (2021) models based on AU data. Additionally, self-supervised models on EEG data (SSUL-*) performed better than fully supervised FSUL-*, validating the success of self-supervised pre-training. Additional layers to the deep model did not improve the performance.

**Table 3:** Self-supervised SSUL-* and fully supervised FSUL-* downstream task performances on EEG data using *H*_*EEG*_ branch.

**Models**	**AUC**	**F1**	**Acc (%)**
SSUL-A	0.79 ± 0.00	0.79 ± 0.00	79.04 ± 0.46
SSUL-B	0.75 ± 0.01	0.76 ± 0.01	74.91 ± 0.73
SSUL-C	0.70 ± 0.01	0.70 ± 0.01	70.24 ± 0.52
FSUL-A	0.74 ± 0.03	0.73 ± 0.03	73.99 ± 3.37
FSUL-B	0.66 ± 0.03	0.64 ± 0.04	66.41 ± 3.41
FSUL-C	0.70 ± 0.02	0.71 ± 0.03	69.48 ± 2.07

### 4.1. Ablation studies

In order to evaluate the efficacy of the multimodal downstream task with less labeled data, we utilize weighted ensemble and embedding fusion methods to evaluate the correlations between *Z*_*AU*_ and *Z*_*EEG*_. Table 4 summarizes the performance of training *H*_*AU*_ and *H*_*EEG*_ together by fusing *Z*_*AU*_ and *Z*_*EEG*_ as one 1 × 128 embedding for downstream optimization. Fusing the embeddings together will force the network to learn a joint distribution of the embeddings. Due to the small amount of labeled data and the complex non-linear spatio-temporal embeddings of both AU and EEG, the embedding fusion model underperforms compared to the unimodal methods while the multimodal weighted ensemble method outperforms both EEG and AU models.

**Table 4:** Downstream task performance for embedding fusion optimization for SSML-A MLP instead of weighted ensemble.

**Performance**	**Embedding fusion**	**Weighted ensemble**
AUC	0.74 ± 0.01	0.81 ± 0.01
F1	0.73 ± 0.02	0.79 ± 0.02
Acc	74.00 ± 1.24 %	80.80 ± 2.36 %

Multimodal weighted ensemble outperforms the embedding fusion method.

### 4.2. Impact of upper and lower facial muscles

**Figure 4:**
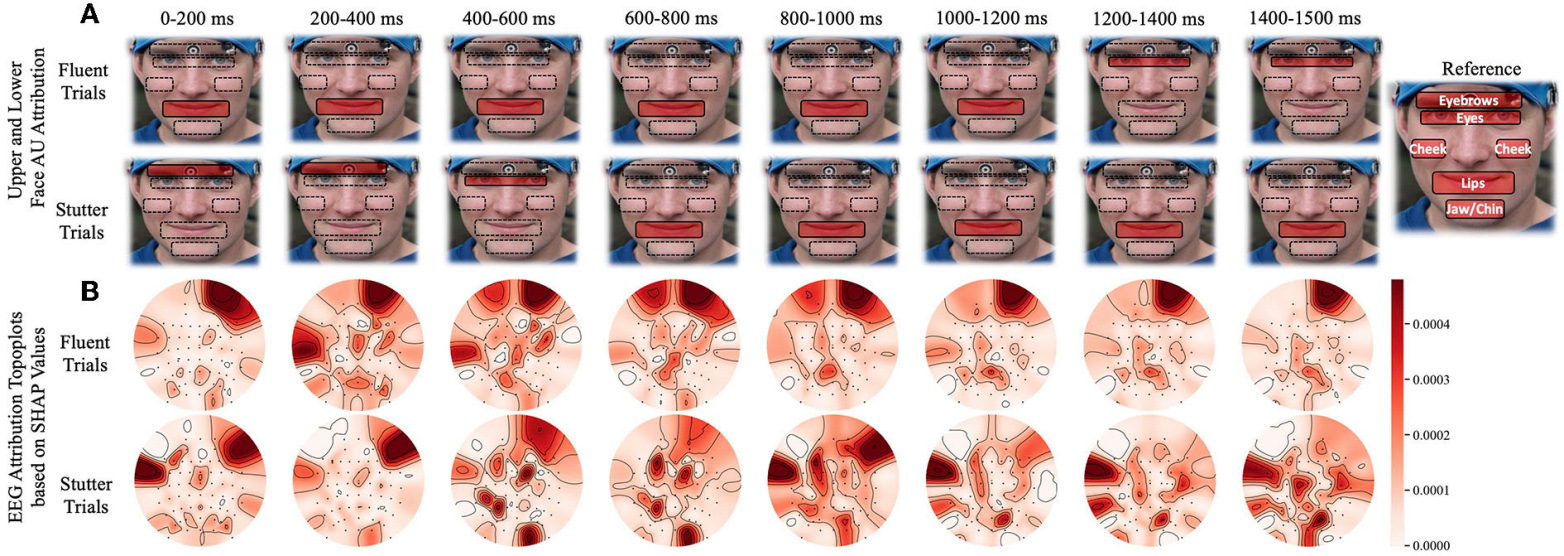
Time window analysis of facial muscle movement and EEG activity with respect to predictions of fluent vs. stuttered trials. **(A)** Facial muscle movement attribution regions in the upper and lower face. The red highlighted regions show the areas with the most predictability in the upper or lower face. **(B)** Topographic plots showing levels of predictability of EEG signals (Shapley values). Circular topographic plots: up, frontal electrode sites; down, posterior sites, left and right correspond to the left and right scalp sites. AU, attribution unit; SHAP, Shapley.

As the subject progresses from speech preparation to production (S1 to S2), the upper and lower facial regions have different temporal patterns that distinguish fluent vs. stuttered trials. As Figure 4A shows, we found a considerable statistical correlation of both upper (*F* = 10.54, *p* < 0.005) and lower (*F* = 69.96, *p* < 0.001) facial muscles toward predicting fluent vs. stuttered trials. These predictions were driven by attributions peaking at different times between S1 and S2. Specifically, the upper facial muscles related to inner brow raiser (AU1, *F* = 56.4, *p* < 0.001), outer brow raiser (AU2, *F* = 56.6, *p* < 0.001), brow lowerer (AU4, *F* = 25.76, *p* < 0.005), and cheek raiser (AU6, *F* = 32.27, *p* < 0.005) peaked after S1 for stuttered trials but peaked before S2 for the fluent trials. The opposite pattern occurred in the lower facial region with dimpler (AU14, *F* = 93.3, *p* < 0.001), lip corner depressor (AU15, *F* = 25.21, *p* < 0.005), and lip stretcher (AU20, *F* = 91.45, *p* < 0.001) peaking after S1 for fluent trials and before S2 for stuttered trials.

### 4.3. Impact of brain regions

**Figure 5:**
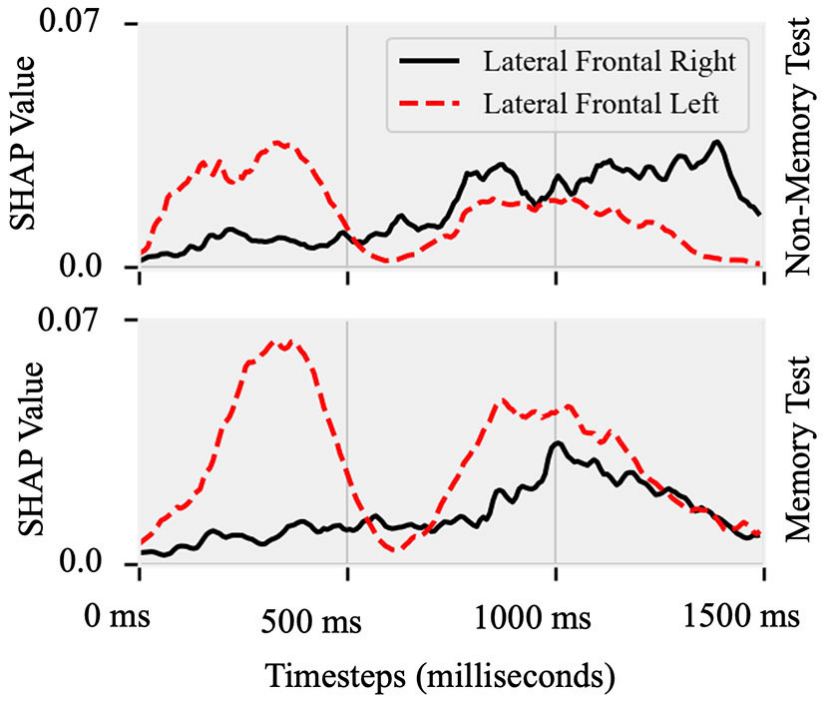
Shapley values of EEG recorded from lateral-frontal left and right hemispheres Topographic plot as a function of time between the cue (0 ms) and target (1,500 ms) onset in the Non-Memory and Memory test paradigms. In both paradigms, predictability of stuttered or fluent speech was maximal after S1 onset at the left site and closer to S2 onset at the right hemisphere site.

*Attributions show hemispheric differences in the brain states that result in either fluent or stuttered speech output*. As illustrated in Figure 4B, EEG signals from the anterior frontal (AF, *F* = 79.10, *p* < 0.001), lateral frontal (LF, *F* = 15.45, *p* < 0.001), and central (C, *F* = 9.90, *p* < 0.005) electrodes were highly significant at predicting fluent vs. stuttered trial. Specifically, the right frontal region (FP2, *F* = 27.04, *p* < 0.001) was related to predicting a fluent trial while the left temporal region (T7, *F* = 29.07, *p* < 0.005) was related to predicting a stuttered trial. These results suggest that the left temporal region attributes toward predicting a future stuttered trial while the right frontal region swings between attributions which contribute to a fluent or a stuttered trial as illustrated in Figure 5.

### 4.4. Improvements with multimodal training

Unimodal training from scratch on EEG and AU data is always attributed to anterior frontal electrodes FPZ and FP1 in the brain and brow lowerer (AU4), cheek raiser (AU6), and dimpler (AU14) in the face. This means that the model always uses a few channels of information to make the prediction. SSUL on EEG and AU improved the results slightly. SSML using the weighted ensemble method generated high attributing AUs from both upper and lower face and EEG channels from frontal, temporal, and parietal regions seen in AWS. Additionally, we see consistent temporal behavior related to speech preparation (Brown et al., 2005; Kell et al., 2009; Chang et al., 2019) in AWS which emphasizes that our multimodal latent space learned to map feature-wise correlations between modalities.

There are several reasons why the success of AI prediction using both EEG and facial signals is unlikely due to contamination of the EEG signal by facial EMG. First, the two signals are in different frequency bands (EEG low frequency slow waves, EMG high frequency muscle activity). Second, pre-processing removed EMG artifacts from EEG. Lastly, left and right frontal electrodes had different prediction patterns based on Shapley attribution, while facial muscle activity was symmetrical. A weighted ensemble of self-supervised pre-trained *H*_*AU*_ and *H*_*EEG*_ using Equation 5 forces the network to generalize and correlate facial microexpressions and corresponding brain activity over time. This ensemble-learning using pre-trained networks reduces the effect of overfitting of the overall multimodal network to either the facial expressions or EEG signals which can be verified by the low variance of performance statistics in Table 2 for the 10-fold cross-validation tests.

### 4.5. Theoretical considerations

We predicted that brain dynamics associated with speech fluency may be evident by peripheral muscle activity, which would act as an indicator of brain activity. Similar psychophysiological approaches index brain activity with autonomic nervous system measures, such as skin conductance responses and level, pupil size, heart rate variability but also include electromyographic measures (Cacioppo et al., 2016). The present observations provide a new line of support for the idea that stuttering is, in part, due to aberrant motor control during speech preparation that occurs well before the stuttering behavior itself. Our AI methods to define facial muscle activity were capable of accurately predicting speech behavior up to about 4 s later (1.5 s from S1 to S2, with a speech onset latency up to about 2.5 s from S2).

We can only lightly speculate on the mechanisms that relate facial muscle activity patterns to the speech in AWS. There is a neuroanatomical basis for why facial muscles may show different activation patterns before fluent vs. stuttered speech. Facial areas of the motor cortex homunculus are located right next to areas controlling speech articulators. Classic ideas suggest that “motor overflow” is a general phenomenon in neurological disorders, whereby imprecise motor control at the level of the primary motor cortex activates regions of the homunculus beyond the intended ones (Hoy et al., 2004). Research on stuttering has found that motor cortex dysfunction can be evident in non-speech cortical areas (Kiziltan and Akalin, 1996; Chang et al., 2019). AWS also show deficits in motor sequence learning (Smits-Bandstra et al., 2006). It is also worth noting that feedback from facial EMG muscle activity patterns can be helpful in speech therapy (Hancock et al., 1998).

There is substantial MRI literature examining hemispheric differences in people who stutter. The focus is on left frontal speech and motor areas and their homologous right hemisphere structures [reviewed in (Smith and Weber, 2017; Chang et al., 2019)]. The most consistent finding is a decrease in white matter integrity along the left hemisphere superior longitudinal fasciculus, which connects frontal speech/language and posterior auditory areas. In concert, left frontal regions (inferior frontal gyrus, ventral premotor) show irregular gray matter development in people who stutter (Beal et al., 2015), consistent with broader fronto-occipital asymmetry differences (Mock et al., 2012). White matter integrity in the left orbitofrontal gyrus is also reduced and greater in those who recovered from stuttering as children (Kell et al., 2009). People who stutter show increased right frontal hemodynamic responses along with reduced left auditory responses (Brown et al., 2005; Budde et al., 2014), which normalize under conditions that enhance speech fluency (De Nil et al., 2003; Lu et al., 2017). One limitation of this line of work is that MRI measures have limited temporal resolution, while EEG can image neural activity with millisecond-level precision. This study tracked rapid brain dynamics in left and right frontal areas and found that both regions contributed to predicting fluent or stuttered speech, with left frontal areas being predictive early in each trial and predictive right hemisphere activity occurring later. Such patterns are informative for understanding brain mechanisms that drive stuttering in real-time.

### 4.6. Limitations and future work

This project did not attempt to test the generalizability of the current multimodal network to a different cohort of AWS subjects, children who stutter, or to different test conditions. Relevant variables for test conditions include the specific speaking tasks and recording parameters for EEG (e.g., number of channels and locations, data processing) and facial video (camera, lighting conditions). However, the proposed self-supervised learning paradigm clearly shows improvements compared to unimodal counterparts and other fully supervised methods, showing that our SSML-* architectures are the current state-of-the-art models in controlled experimental settings for AWS across a substantial range of severities. Another limitation is that there was not enough data to individually examine subtypes of disfluency (prolongations, silent blocks, part-word repetition). Thus, potential differences in neural and facial muscle activities during speech preparation that culminate in a block vs. a prolongation, for example, could not be identified.

Future work could test the hypothesis that lower facial muscle activity occurring shortly before the cue (S2) to speak reflects speech motor programming. The possibility that the transient, early activity in upper facial muscles previously associated with longer-lasting affect states is also subject to testing. Future work can explore a generalized few-shot subject-wise self-supervision algorithm that could rapidly adapt the trained models for personalized adjunct to speech therapy. Testing with more participants would also help to better understand the role of individual differences relative to commonalities across individuals. The current study is focused on stuttering disfluency. However, for many similar neurological disorders that also have fluctuating brain states, such as stroke, Alzheimer's disease, aphasia, Parkinson's disease, and epilepsy, domain adaptation may be used to generalize the method. We do not foresee any negative societal impacts as a consequence of our work.

## 5. Conclusion

In this paper, we designed a self-supervised multimodal explanation algorithm that successfully correlate fluctuating brain states of AWS and facial muscle activity quantified as facial AUs. By forcing the latent space of external behavior (face) using the internal brain-states (EEG), we learn both microexpression and cognitive state representations. By carrying out multimodal training of EEG and AU modalities and explaining the predictions using the proposed multimodal Shapley explainer, we found correlations among upper and lower facial regions to left-temporal and bilateral frontal brain regions. Such correlations were evident at specific time windows, without a priori labeled knowledge about human speech behavior in AWS provided to the neural network. Our explainability method identified key regions from both face and brain that may drive the production of either fluent or stuttered speech in AWS. The brain regions identified are also the same regions that differ both structurally and functionally between people who stutter and fluent controls (Brown et al., 2005). The proposed methodology can be applied for multimodal speech therapies and also generalized to other neurological disorders with fluctuating brain states, such as stroke or Alzheimer's disease.

## Data availability statement

Requests for resources, reagents, and any further queries should be directed to and will be fulfilled by Peyman Najafirad (peyman.najafirad@utsa.edu) and Edward Golob (edward.golob@utsa.edu) in accordance with the IRB protocol.

## Ethics statement

The studies involving human participants were reviewed and approved by the University of Texas at San Antonio Institutional Review Board (UTSA IRB, IRB #17-097, Cognitive Potentials, Approval Date: 3-30-2017), consistent with the Declaration of Helsinki. The patients/participants provided their written informed consent to participate in this study. Written informed consent was obtained from the individual(s) for the publication of any potentially identifiable images or data included in this article.

## Author contributions

AD and PN designed the multimodal deep learning architecture and multimodal explanation algorithm. AD implemented the framework and ran the experiments. JM and EG designed the cognitive experiment where the data were collected, the deep learning model was evaluated, and the neuroscience interpretations were provided. FI helped with stimulus selection, participant recruitment, speech and stuttering assessment, and coding. JM, EG, and YH helped interpret the findings focused on the impact of brain regions, EEG signal analysis, and multimodal explanations. All authors contributed to the article and approved the submitted version.

## Funding

This work was partly supported by the National Institutes of Health (NIH) under Grant DC016353 and the Open Cloud Institute (OCI) at the University of Texas at San Antonio (UTSA).

## Conflict of interest

The authors declare that the research was conducted in the absence of any commercial or financial relationships that could be construed as a potential conflict of interest.

## Publisher's note

All claims expressed in this article are solely those of the authors and do not necessarily represent those of their affiliated organizations, or those of the publisher, the editors and the reviewers. Any product that may be evaluated in this article, or claim that may be made by its manufacturer, is not guaranteed or endorsed by the publisher.
